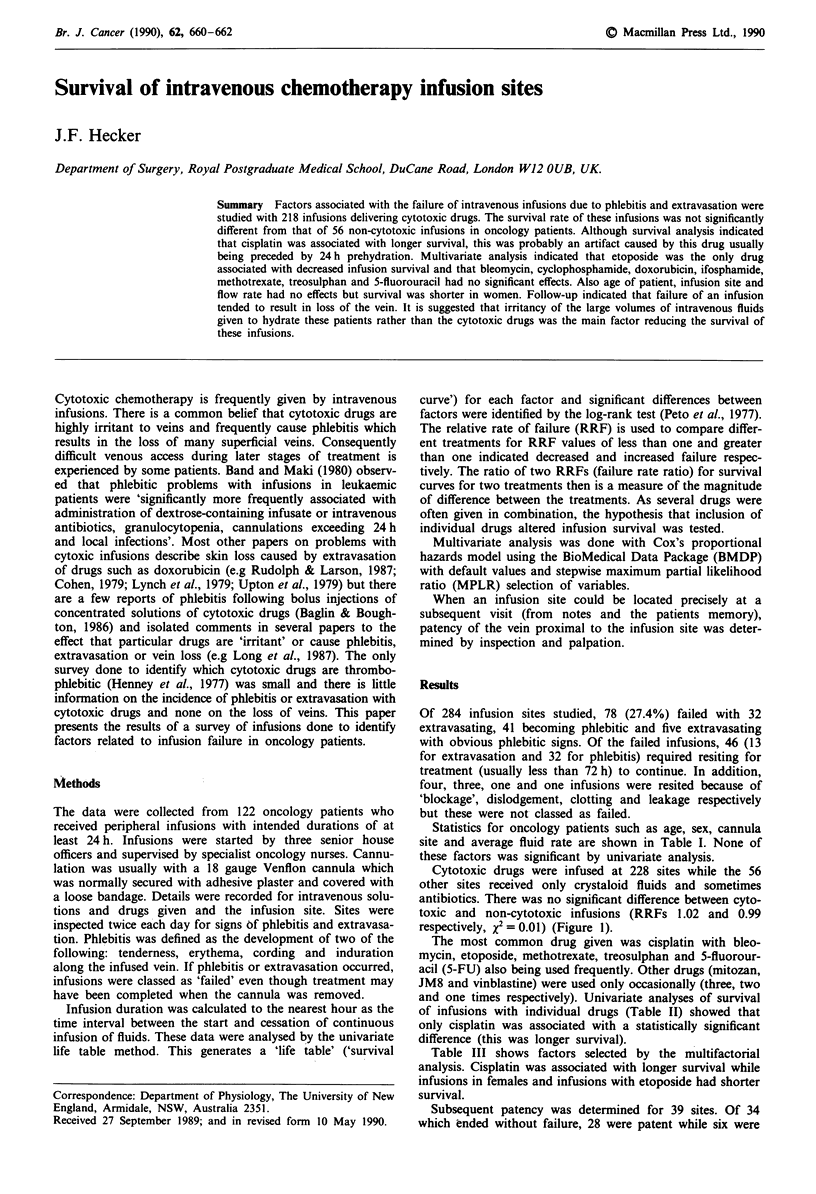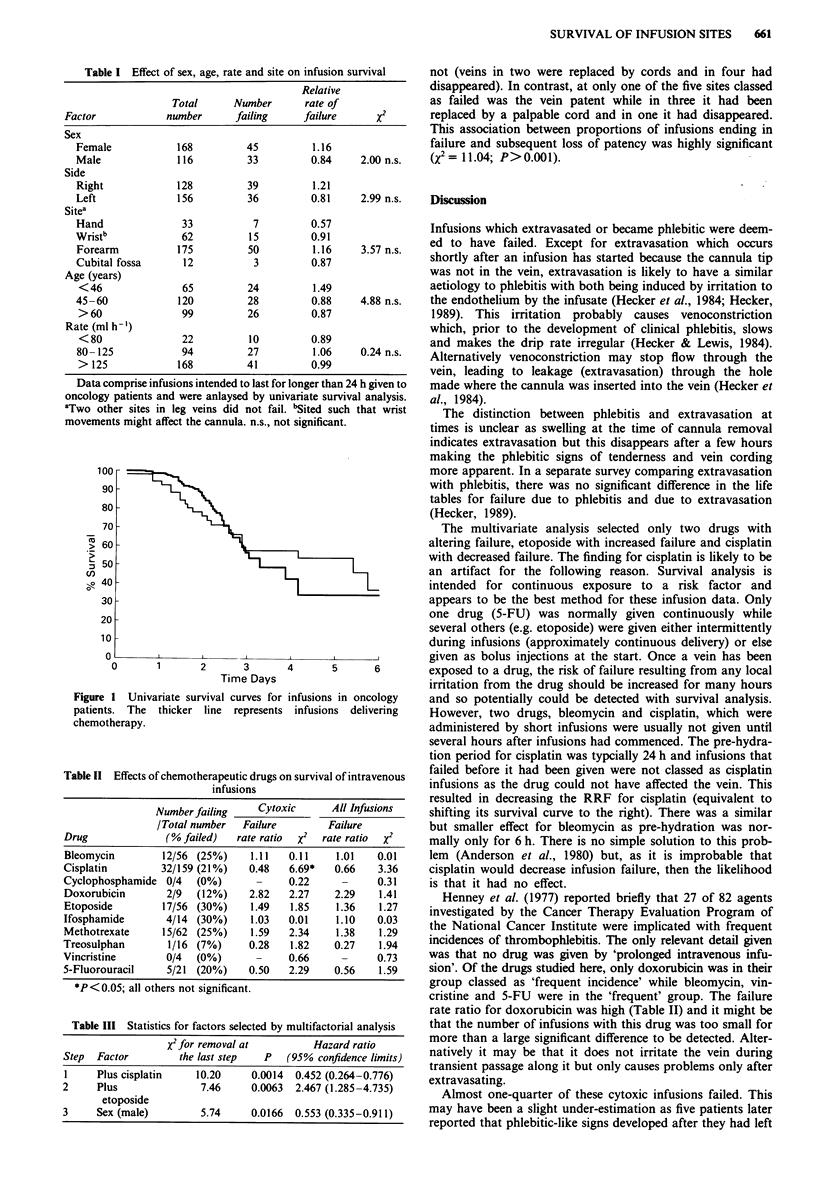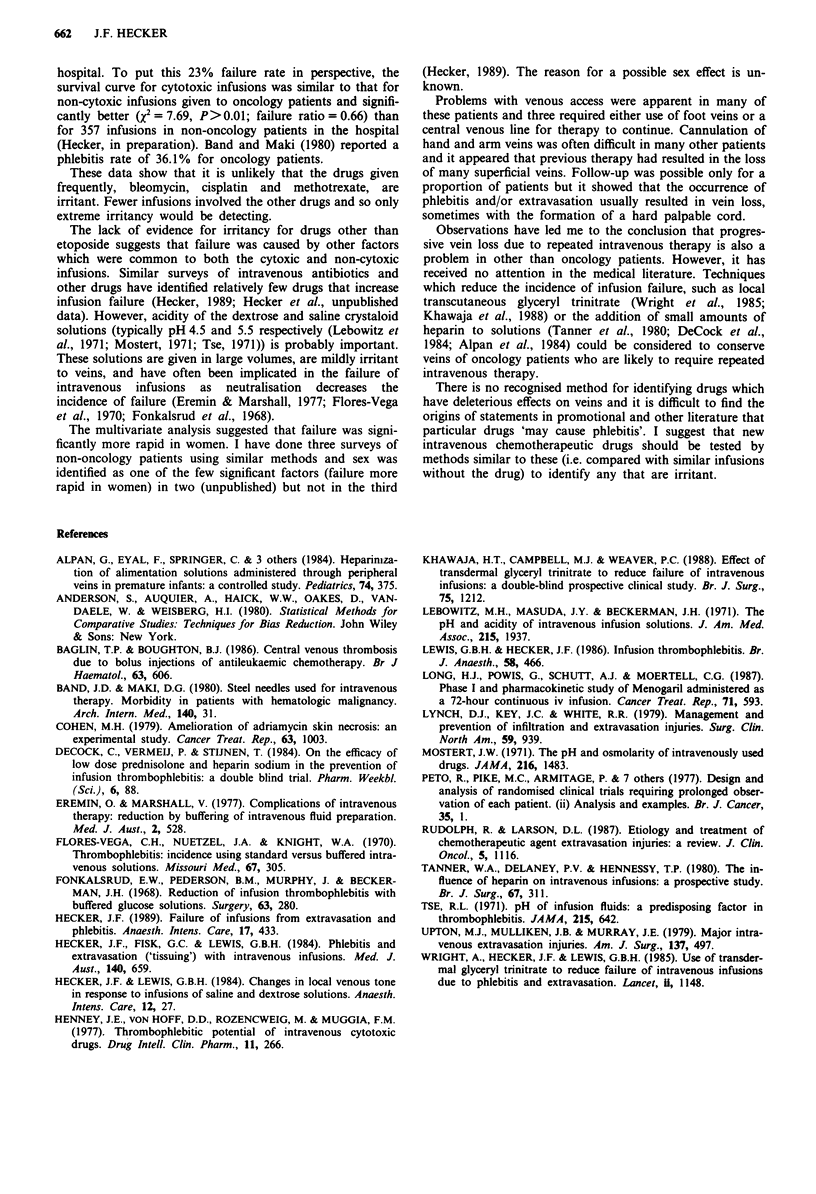# Survival of intravenous chemotherapy infusion sites.

**DOI:** 10.1038/bjc.1990.351

**Published:** 1990-10

**Authors:** J. F. Hecker

**Affiliations:** Department of Surgery, Royal Postgraduate Medical School, London, UK.

## Abstract

Factors associated with the failure of intravenous infusions due to phlebitis and extravasation were studied with 218 infusions delivering cytotoxic drugs. The survival rate of these infusions was not significantly different from that of 56 non-cytotoxic infusions in oncology patients. Although survival analysis indicated that cisplatin was associated with longer survival, this was probably an artifact caused by this drug usually being preceded by 24 h prehydration. Multivariate analysis indicated that etoposide was the only drug associated with decreased infusion survival and that bleomycin, cyclophosphamide, doxorubicin, ifosphamide, methotrexate, treosulphan and 5-fluorouracil had no significant effects. Also age of patient, infusion site and flow rate had no effects but survival was shorter in women. Follow-up indicated that failure of an infusion tended to result in loss of the vein. It is suggested that irritancy of the large volumes of intravenous fluids given to hydrate these patients rather than the cytotoxic drugs was the main factor reducing the survival of these infusions.


 © Macmillan Press Ltd., 1990

### Survival of intravenous chemotherapy infusion sites

J.F. Hecker

*Department of Surgery, Royal Postgraduate Medical School, DuCane Road, London W12 0UB, UK.*

**Summary** Factors associated with the failure of intravenous infusions due to phlebitis and extravasation were studied with 218 infusions delivering cytotoxic drugs. The survival rate of these infusions was not significantly different from that of 56 non-cytotoxic infusions in oncology patients. Although survival analysis indicated that cisplatin was associated with longer survival, this was probably an artifact caused by this drug usually being preceded by 24 h prehydration. Multivariate analysis indicated that etoposide was the only drug associated with decreased infusion survival and that bleomycin, cyclophosphamide, doxorubicin, ifosphamide, methotrexate, treosulphan and 5-fluorouracil had no significant effects. Also age of patient, infusion site and flow rate had no effects but survival was shorter in women. Follow-up indicated that failure of an infusion tended to result in loss of the vein. It is suggested that irritancy of the large volumes of intravenous fluids given to hydrate these patients rather than the cytotoxic drugs was the main factor reducing the survival of these infusions.

Cytotoxic chemotherapy is frequently given by intravenous infusions. There is a common belief that cytotoxic drugs are highly irritant to veins and frequently cause phlebitis which results in the loss of many superficial veins. Consequently difficult venous access during later stages of treatment is experienced by some patients. Band and Maki (1980) observed that phlebitic problems with infusions in leukaemic patients were 'significantly more frequently associated with administration of dextrose-containing infusate or intravenous antibiotics, granulocytopenia, cannulations exceeding 24 h and local infections'. Most other papers on problems with cytoxic infusions describe skin loss caused by extravasation of drugs such as doxorubicin (e.g Rudolph & Larson, 1987; Cohen, 1979; Lynch *et al.*, 1979; Upton *et al.*, 1979) but there are a few reports of phlebitis following bolus injections of concentrated solutions of cytotoxic drugs (Baglin & Boughton, 1986) and isolated comments in several papers to the effect that particular drugs are 'irritant' or cause phlebitis, extravasation or vein loss (e.g Long *et al.*, 1987). The only survey done to identify which cytotoxic drugs are thrombophlebitic (Henney *et al.*, 1977) was small and there is little information on the incidence of phlebitis or extravasation with cytotoxic drugs and none on the loss of veins. This paper presents the results of a survey of infusions done to identify factors related to infusion failure in oncology patients.

### Methods

The data were collected from 122 oncology patients who received peripheral infusions with intended durations of at least 24 h. Infusions were started by three senior house officers and supervised by specialist oncology nurses. Cannulation was usually with a 18 gauge Venflon cannula which was normally secured with adhesive plaster and covered with a loose bandage. Details were recorded for intravenous solutions and drugs given and the infusion site. Sites were inspected twice each day for signs of phlebitis and extravasation. Phlebitis was defined as the development of two of the following: tenderness, erythema, cording and induration along the infused vein. If phlebitis or extravasation occurred, infusions were classed as 'failed' even though treatment may have been completed when the cannula was removed.

Infusion duration was calculated to the nearest hour as the time interval between the start and cessation of continuous infusion of fluids. These data were analysed by the univariate life table method. This generates a 'life table' ('survival curve') for each factor and significant differences between factors were identified by the log-rank test (Peto *et al.*, 1977). The relative rate of failure (RRF) is used to compare different treatments for RRF values of less than one and greater than one indicated decreased and increased failure respectively. The ratio of two RRFs (failure rate ratio) for survival curves for two treatments then is a measure of the magnitude of difference between the treatments. As several drugs were often given in combination, the hypothesis that inclusion of individual drugs altered infusion survival was tested.

Multivariate analysis was done with Cox's proportional hazards model using the BioMedical Data Package (BMDP) with default values and stepwise maximum partial likelihood ratio (MPLR) selection of variables.

When an infusion site could be located precisely at a subsequent visit (from notes and the patients memory), patency of the vein proximal to the infusion site was determined by inspection and palpation.

### Results

Of 284 infusion sites studied, 78 (27.4%) failed with 32 extravasating, 41 becoming phlebitic and five extravasating with obvious phlebitic signs. Of the failed infusions, 46 (13 for extravasation and 32 for phlebitis) required resiting for treatment (usually less than 72 h) to continue. In addition, four, three, one and one infusions were resited because of 'blockage', dislodgement, clotting and leakage respectively but these were not classed as failed.

Statistics for oncology patients such as age, sex, cannula site and average fluid rate are shown in Table I. None of these factors was significant by univariate analysis.

Cytotoxic drugs were infused at 228 sites while the 56 other sites received only crystaloid fluids and sometimes antibiotics. There was no significant difference between cytotoxic and non-cytotoxic infusions (RRFs 1.02 and 0.99 respectively, $\chi^2 = 0.01$) (Figure 1).

The most common drug given was cisplatin with bleomycin, etoposide, methotrexate, treosulphan and 5-fluorouracil (5-FU) also being used frequently. Other drugs (mitozan, JM8 and vinblastine) were used only occasionally (three, two and one times respectively). Univariate analyses of survival of infusions with individual drugs (Table II) showed that only cisplatin was associated with a statistically significant difference (this was longer survival).

Table III shows factors selected by the multifactorial analysis. Cisplatin was associated with longer survival while infusions in females and infusions with etoposide had shorter survival.

Subsequent patency was determined for 39 sites. Of 34 which ended without failure, 28 were patent while six were

Correspondence: Department of Physiology, The University of New England, Armidale, NSW, Australia 2351.
Received 27 September 1989; and in revised form 10 May 1990.

**Table I** Effect of sex, age, rate and site on infusion survival

| Factor | Total number | Number failing | Relative rate of failure | $\chi^2$ |
|---|---|---|---|---|
| Sex | | | | |
| Female | 168 | 45 | 1.16 | |
| Male | 116 | 33 | 0.84 | 2.00 n.s. |
| Side | | | | |
| Right | 128 | 39 | 1.21 | |
| Left | 156 | 36 | 0.81 | 2.99 n.s. |
| Site[a] | | | | |
| Hand | 33 | 7 | 0.57 | |
| Wrist[b] | 62 | 15 | 0.91 | |
| Forearm | 175 | 50 | 1.16 | 3.57 n.s. |
| Cubital fossa | 12 | 3 | 0.87 | |
| Age (years) | | | | |
| <46 | 65 | 24 | 1.49 | |
| 45-60 | 120 | 28 | 0.88 | 4.88 n.s. |
| >60 | 99 | 26 | 0.87 | |
| Rate (ml $h^{-1}$) | | | | |
| <80 | 22 | 10 | 0.89 | |
| 80-125 | 94 | 27 | 1.06 | 0.24 n.s. |
| >125 | 168 | 41 | 0.99 | |

Data comprise infusions intended to last for longer than 24 h given to oncology patients and were anlaysed by univariate survival analysis. [a]Two other sites in leg veins did not fail. [b]Sited such that wrist movements might affect the cannula. n.s., not significant.

**Figure 1** Univariate survival curves for infusions in oncology patients. The thicker line represents infusions delivering chemotherapy.

**Table II** Effects of chemotherapeutic drugs on survival of intravenous infusions

| Drug | Number failing /Total number (% failed) | Cytoxic Failure rate ratio | Cytoxic $\chi^2$ | All Infusions Failure rate ratio | All Infusions $\chi^2$ |
|---|---|---|---|---|---|
| Bleomycin | 12/56 (25%) | 1.11 | 0.11 | 1.01 | 0.01 |
| Cisplatin | 32/159 (21%) | 0.48 | 6.69* | 0.66 | 3.36 |
| Cyclophosphamide | 0/4 (0%) | - | 0.22 | - | 0.31 |
| Doxorubicin | 2/9 (12%) | 2.82 | 2.27 | 2.29 | 1.41 |
| Etoposide | 17/56 (30%) | 1.49 | 1.85 | 1.36 | 1.27 |
| Ifosphamide | 4/14 (30%) | 1.03 | 0.01 | 1.10 | 0.03 |
| Methotrexate | 15/62 (25%) | 1.59 | 2.34 | 1.38 | 1.29 |
| Treosulphan | 1/16 (7%) | 0.28 | 1.82 | 0.27 | 1.94 |
| Vincristine | 0/4 (0%) | - | 0.66 | - | 0.73 |
| 5-Fluorouracil | 5/21 (20%) | 0.50 | 2.29 | 0.56 | 1.59 |

*$P<0.05$; all others not significant.

**Table III** Statistics for factors selected by multifactorial analysis

| Step | Factor | $\chi^2$ for removal at the last step | P | Hazard ratio (95% confidence limits) |
|---|---|---|---|---|
| 1 | Plus cisplatin | 10.20 | 0.0014 | 0.452 (0.264-0.776) |
| 2 | Plus etoposide | 7.46 | 0.0063 | 2.467 (1.285-4.735) |
| 3 | Sex (male) | 5.74 | 0.0166 | 0.553 (0.335-0.911) |

not (veins in two were replaced by cords and in four had disappeared). In contrast, at only one of the five sites classed as failed was the vein patent while in three it had been replaced by a palpable cord and in one it had disappeared. This association between proportions of infusions ending in failure and subsequent loss of patency was highly significant ($\chi^2 = 11.04$; $P>0.001$).

### Discussion

Infusions which extravasated or became phlebitic were deemed to have failed. Except for extravasation which occurs shortly after an infusion has started because the cannula tip was not in the vein, extravasation is likely to have a similar aetiology to phlebitis with both being induced by irritation to the endothelium by the infusate (Hecker *et al.*, 1984; Hecker, 1989). This irritation probably causes venoconstriction which, prior to the development of clinical phlebitis, slows and makes the drip rate irregular (Hecker & Lewis, 1984). Alternatively venoconstriction may stop flow through the vein, leading to leakage (extravasation) through the hole made where the cannula was inserted into the vein (Hecker *et al.*, 1984).

The distinction between phlebitis and extravasation at times is unclear as swelling at the time of cannula removal indicates extravasation but this disappears after a few hours making the phlebitic signs of tenderness and vein cording more apparent. In a separate survey comparing extravasation with phlebitis, there was no significant difference in the life tables for failure due to phlebitis and due to extravasation (Hecker, 1989).

The multivariate analysis selected only two drugs with altering failure, etoposide with increased failure and cisplatin with decreased failure. The finding for cisplatin is likely to be an artifact for the following reason. Survival analysis is intended for continuous exposure to a risk factor and appears to be the best method for these infusion data. Only one drug (5-FU) was normally given continuously while several others (e.g. etoposide) were given either intermittently during infusions (approximately continuous delivery) or else given as bolus injections at the start. Once a vein has been exposed to a drug, the risk of failure resulting from any local irritation from the drug should be increased for many hours and so potentially could be detected with survival analysis. However, two drugs, bleomycin and cisplatin, which were administered by short infusions were usually not given until several hours after infusions had commenced. The pre-hydration period for cisplatin was typcially 24 h and infusions that failed before it had been given were not classed as cisplatin infusions as the drug could not have affected the vein. This resulted in decreasing the RRF for cisplatin (equivalent to shifting its survival curve to the right). There was a similar but smaller effect for bleomycin as pre-hydration was normally only for 6 h. There is no simple solution to this problem (Anderson *et al.*, 1980) but, as it is improbable that cisplatin would decrease infusion failure, then the likelihood is that it had no effect.

Henney *et al.* (1977) reported briefly that 27 of 82 agents investigated by the Cancer Therapy Evaluation Program of the National Cancer Institute were implicated with frequent incidences of thrombophlebitis. The only relevant detail given was that no drug was given by 'prolonged intravenous infusion'. Of the drugs studied here, only doxorubicin was in their group classed as 'frequent incidence' while bleomycin, vincristine and 5-FU were in the 'frequent' group. The failure rate ratio for doxorubicin was high (Table II) and it might be that the number of infusions with this drug was too small for more than a large significant difference to be detected. Alternatively it may be that it does not irritate the vein during transient passage along it but only causes problems only after extravasating.

Almost one-quarter of these cytoxic infusions failed. This may have been a slight under-estimation as five patients later reported that phlebitic-like signs developed after they had left hospital. To put this 23% failure rate in perspective, the survival curve for cytotoxic infusions was similar to that for non-cytoxic infusions given to oncology patients and significantly better ($\chi^2 = 7.69$, $P > 0.01$; failure ratio = 0.66) than for 357 infusions in non-oncology patients in the hospital (Hecker, in preparation). Band and Maki (1980) reported a phlebitis rate of 36.1% for oncology patients.

These data show that it is unlikely that the drugs given frequently, bleomycin, cisplatin and methotrexate, are irritant. Fewer infusions involved the other drugs and so only extreme irritancy would be detecting.

The lack of evidence for irritancy for drugs other than etoposide suggests that failure was caused by other factors which were common to both the cytoxic and non-cytoxic infusions. Similar surveys of intravenous antibiotics and other drugs have identified relatively few drugs that increase infusion failure (Hecker, 1989; Hecker *et al.*, unpublished data). However, acidity of the dextrose and saline crystaloid solutions (typically pH 4.5 and 5.5 respectively (Lebowitz *et al.*, 1971; Mostert, 1971; Tse, 1971)) is probably important. These solutions are given in large volumes, are mildly irritant to veins, and have often been implicated in the failure of intravenous infusions as neutralisation decreases the incidence of failure (Eremin & Marshall, 1977; Flores-Vega *et al.*, 1970; Fonkalsrud *et al.*, 1968).

The multivariate analysis suggested that failure was significantly more rapid in women. I have done three surveys of non-oncology patients using similar methods and sex was identified as one of the few significant factors (failure more rapid in women) in two (unpublished) but not in the third (Hecker, 1989). The reason for a possible sex effect is unknown.

Problems with venous access were apparent in many of these patients and three required either use of foot veins or a central venous line for therapy to continue. Cannulation of hand and arm veins was often difficult in many other patients and it appeared that previous therapy had resulted in the loss of many superficial veins. Follow-up was possible only for a proportion of patients but it showed that the occurrence of phlebitis and/or extravasation usually resulted in vein loss, sometimes with the formation of a hard palpable cord.

Observations have led me to the conclusion that progressive vein loss due to repeated intravenous therapy is also a problem in other than oncology patients. However, it has received no attention in the medical literature. Techniques which reduce the incidence of infusion failure, such as local transcutaneous glyceryl trinitrate (Wright *et al.*, 1985; Khawaja *et al.*, 1988) or the addition of small amounts of heparin to solutions (Tanner *et al.*, 1980; DeCock *et al.*, 1984; Alpan *et al.*, 1984) could be considered to conserve veins of oncology patients who are likely to require repeated intravenous therapy.

There is no recognised method for identifying drugs which have deleterious effects on veins and it is difficult to find the origins of statements in promotional and other literature that particular drugs 'may cause phlebitis'. I suggest that new intravenous chemotherapeutic drugs should be tested by methods similar to these (i.e. compared with similar infusions without the drug) to identify any that are irritant.

### References

ALPAN, G., EYAL, F., SPRINGER, C. & 3 others (1984). Heparinization of alimentation solutions administered through peripheral veins in premature infants: a controlled study. *Pediatrics*, **74**, 375.

ANDERSON, S., AUQUIER, A., HAICK, W.W., OAKES, D., VANDAELE, W. & WEISBERG, H.I. (1980). *Statistical Methods for Comparative Studies: Techniques for Bias Reduction*. John Wiley & Sons: New York.

BAGLIN, T.P. & BOUGHTON, B.J. (1986). Central venous thrombosis due to bolus injections of antileukaemic chemotherapy. *Br J Haematol.*, **63**, 606.

BAND, J.D. & MAKI, D.G. (1980). Steel needles used for intravenous therapy. Morbidity in patients with hematologic malignancy. *Arch. Intern. Med.*, **140**, 31.

COHEN, M.H. (1979). Amelioration of adriamycin skin necrosis: an experimental study. *Cancer Treat. Rep.*, **63**, 1003.

DECOCK, C., VERMEIJ, P. & STIJNEN, T. (1984). On the efficacy of low dose prednisolone and heparin sodium in the prevention of infusion thrombophlebitis: a double blind trial. *Pharm. Weekbl. (Sci.)*, **6**, 88.

EREMIN, O. & MARSHALL, V. (1977). Complications of intravenous therapy: reduction by buffering of intravenous fluid preparation. *Med. J. Aust.*, **2**, 528.

FLORES-VEGA, C.H., NUETZEL, J.A. & KNIGHT, W.A. (1970). Thrombophlebitis: incidence using standard versus buffered intravenous solutions. *Missouri Med.*, **67**, 305.

FONKALSRUD, E.W., PEDERSON, B.M., MURPHY, J. & BECKERMAN, J.H. (1968). Reduction of infusion thrombophlebitis with buffered glucose solutions. *Surgery*, **63**, 280.

HECKER, J.F. (1989). Failure of infusions from extravasation and phlebitis. *Anaesth. Intens. Care*, **17**, 433.

HECKER, J.F., FISK, G.C. & LEWIS, G.B.H. (1984). Phlebitis and extravasation ('tissuing') with intravenous infusions. *Med. J. Aust.*, **140**, 659.

HECKER, J.F. & LEWIS, G.B.H. (1984). Changes in local venous tone in response to infusions of saline and dextrose solutions. *Anaesth. Intens. Care*, **12**, 27.

HENNEY, J.E., VON HOFF, D.D., ROZENCWEIG, M. & MUGGIA, F.M. (1977). Thrombophlebitic potential of intravenous cytotoxic drugs. *Drug Intell. Clin. Pharm.*, **11**, 266.

KHAWAJA, H.T., CAMPBELL, M.J. & WEAVER, P.C. (1988). Effect of transdermal glyceryl trinitrate to reduce failure of intravenous infusions: a double-blind prospective clinical study. *Br. J. Surg.*, **75**, 1212.

LEBOWITZ, M.H., MASUDA, J.Y. & BECKERMAN, J.H. (1971). The pH and acidity of intravenous infusion solutions. *J. Am. Med. Assoc.*, **215**, 1937.

LEWIS, G.B.H. & HECKER, J.F. (1986). Infusion thrombophlebitis. *Br. J. Anaesth.*, **58**, 466.

LONG, H.J., POWIS, G., SCHUTT, A.J. & MOERTELL, C.G. (1987). Phase I and pharmacokinetic study of Menogaril administered as a 72-hour continuous iv infusion. *Cancer Treat. Rep.*, **71**, 593.

LYNCH, D.J., KEY, J.C. & WHITE, R.R. (1979). Management and prevention of infiltration and extravasation injuries. *Surg. Clin. North Am.*, **59**, 939.

MOSTERT, J.W. (1971). The pH and osmolarity of intravenously used drugs. *JAMA*, **216**, 1483.

PETO, R., PIKE, M.C., ARMITAGE, P. & 7 others (1977). Design and analysis of randomised clinical trials requiring prolonged observation of each patient. (ii) Analysis and examples. *Br. J. Cancer*, **35**, 1.

RUDOLPH, R. & LARSON, D.L. (1987). Etiology and treatment of chemotherapeutic agent extravasation injuries: a review. *J. Clin. Oncol.*, **5**, 1116.

TANNER, W.A., DELANEY, P.V. & HENNESSY, T.P. (1980). The influence of heparin on intravenous infusions: a prospective study. *Br. J. Surg.*, **67**, 311.

TSE, R.L. (1971). pH of infusion fluids: a predisposing factor in thrombophlebitis. *JAMA*, **215**, 642.

UPTON, M.J., MULLIKEN, J.B. & MURRAY, J.E. (1979). Major intravenous extravasation injuries. *Am. J. Surg.*, **137**, 497.

WRIGHT, A., HECKER, J.F. & LEWIS, G.B.H. (1985). Use of transdermal glyceryl trinitrate to reduce failure of intravenous infusions due to phlebitis and extravasation. *Lancet*, **ii**, 1148.